# Community-based serological screening for Chagas disease in London: a cross-sectional, observational pilot study

**DOI:** 10.1136/bmjph-2025-002940

**Published:** 2025-10-27

**Authors:** Natalie Elkheir, Liliana Sanchez Zelaya, Laura Valderrama Penagos, Laura Lopez, Rodrigo Ville, Estefania Sienra-Iracheta, Temitope Fisayo, Pat Lok, Fionnuala Ryan, Ana Bayona, Jose Garcia Romero, Clare E Warrell, Nicolas Massie, Cristina Fernandez-Turienzo, Valentina De Sario, Jenna Abaakouk, Karina Guzman Miranda, Amel Alfulaij, Cristina Suarez, Maria-Cristina Loader, Catherine Dominic, Maykon Diego Melo, Marcelle Costa Marinho, Marcela Bojorquez Segura, Heloise Gerber, Anna Bote-Casamitjana, Patricia Miralhes, Alyssa Chase-Vilchez, Declan Crilly, Jaimie Wilson-Goldsmith, Barbara de Barros, Jessica Carter, Helen Liddy, Niamh Murphy, Debbie Nolder, Amaya Bustinduy, Laura E Nabarro, Peter L Chiodini, David Moore

**Affiliations:** 1 Clinical Research Department, London School of Hygiene & Tropical Medicine, London, UK; 2 Hospital for Tropical Diseases, University College London Hospitals, London, UK; 3 UK Chagas Hub, London, UK; 4 Diagnostic Parasitology Laboratory, London School of Hygiene & Tropical Medicine, London, UK

**Keywords:** Public Health, Epidemiology, Community Health

## Abstract

**Introduction:**

Chagas disease (CD) is increasingly recognised as a public health problem in non-endemic settings. The UK is home to a large Latin American migrant population; yet, there is no formal screening programme for CD.

The aim of this study was to co-design and evaluate a community-based screening initiative for CD in Latin American migrants in London, UK.

**Methods:**

This was a cross-sectional, observational pilot study, using questionnaires and point of care tests (POCTs: finger-prick lateral flow assays for qualitative detection of Immunoglobulin G (IgG) to *Trypanosoma cruzi*). Screening was offered at nine community (non-healthcare setting) events advertised on social media and by word-of-mouth. The main outcome measures were seroprevalence of *T. cruzi* infection, by age, sex, country of birth and event type, positive predictive value (PPV) of the POCT, linkage to care and screening yield.

**Results:**

334 adult participants (254 at CD-specific events and 80 at other events) participated in screening between December 2021 and May 2023. All were first-generation or second-generation migrants from South America, Central America or Mexico. 206 (62%) were born in Bolivia and 223 (67%) were women.

A total of 79 out of 334 (24%) participants screened positively, of whom 77 (97%) attended for confirmatory laboratory serology and 70 (21% total population screened) were confirmed as true cases (two laboratory-performed serological assays positive). The POCT-PPV overall was 91%. 90% of the true confirmed cases detected through community screening were still engaged in specialist care at a mean of 2.5 years follow-up. The number needed to screen to link one confirmed case into specialist care was 5.3.

**Conclusions:**

Active case finding through delivery of targeted community-based screening using POCTs at either dedicated events or as pop-up testing at other events can effectively identify people with CD, with a high yield and minimal loss to follow-up.

**WHAT IS ALREADY KNOWN ON THIS TOPIC**

**WHAT THIS STUDY ADDS**

- This is the first study to co-design and evaluate a community-based screening initiative for Chagas disease in the UK. One in five people who were offered screening at community events were positive, most of whom were linked into care for specialist assessment and antiparasitic treatment.

**HOW THIS STUDY MIGHT AFFECT RESEARCH, PRACTICE OR POLICY**

- Based on the data obtained from this study, targeted screening programmes for Chagas disease are needed. This study demonstrated that community-based screening, using point of care tests, is effective at reaching high-prevalence groups and linking people with Chagas disease into specialist care.

## Introduction

 Chagas disease (CD) is a chronic neglected tropical disease endemic in the Americas, caused by the protozoan *Trypanosoma cruzi*.^1^ An estimated seven million people worldwide are infected with this parasite.^2^,^4^ CD is found mainly in rural areas of the 21 continental Latin American countries (Mexico, Central America and South America), where it is principally transmitted by infected blood-feeding triatomine insects (vector-borne transmission)^5^. *T. cruzi* can also be transmitted through mother-to-child (vertical) transmission,^6 7^ oral transmission^8^ and, in the absence of donor screening, through blood transfusion and organ transplantation.^9^ CD was once entirely confined to the Americas; however, through globalisation and migration, it is increasingly recognised as a public health problem among Latin American migrants in Europe and the US.^10^,^13^

CD has an acute phase, which is usually asymptomatic or non-specific, such as presentation with fever and lymphadenopathy and typically goes unrecognised. The acute phase usually resolves spontaneously, after which time individuals remain chronically infected if untreated. Approximately one third of *T. cruzi* seropositive individuals develop end-organ (‘determinate’) disease, 10–30 years after acute infection (with cardiac disease, digestive disease or both). Cardiac determinate disease can manifest as conduction defects, dysrhythmias (a potential cause of sudden cardiac death), ventricular aneurysm and dilated cardiomyopathy.^14^ Gastrointestinal disease includes oesophageal and colonic dysmotility and eventually dilatation—often referred to as mega-oesophagus or mega-colon.^15^ Reactivation in the context of immunosuppression (particularly untreated HIV infection) can lead to high morbidity and mortality if unrecognised.^16^ Early detection of CD, and subsequent trypanocidal therapy, can mitigate the risks of determinate disease and prevent ongoing (vertical) transmission.^1^ Antiparasitic treatment administered to women of reproductive age almost eliminates the risk of vertical transmission in future pregnancies and so this is an important group to consider for screening.^1 6 7^ As the vast majority of infection is asymptomatic, this can only be achieved at scale through screening.

According to the 2021 Census, England and Wales are home to over 280 000 people born in CD-endemic countries, over half of whom live in London.^17^ The actual Latin American population size to be considered for screening is much greater, as people born to mothers from endemic countries (second-generation migrants) are not captured in this data nor are undocumented migrants reliably counted. Women born in CD-endemic countries give birth to 5000 infants each year in the UK.^18^ Despite the availability of screening tests (*T. cruzi* serology) and antiparasitic treatment (including the UK’s only CD-specialist clinic at the Hospital for Tropical Diseases in London), in 2016, it was estimated that 97% of people with *T. cruzi* infection in the UK were undiagnosed.^19^ *T. cruzi* screening has been implemented in blood banks in the UK since 1998.^20^ The UK government’s Migrant Health Guide also recommends screening migrants from endemic areas, in particular, pregnant women and other women of reproductive age;^21^ however, there is no formal migrant or antenatal screening programme nor consensus on how best this might be done, resulting in minimal screening of the at-risk population.

One potential intervention to screen the at-risk population for CD is to use point of care tests (POCTs). There are commercially available POCTs that have performed well in endemic settings such as Argentina and Bolivia (sensitivity and specificity over 92%), with high user—and patient—acceptability demonstrated.^22 23^ Qualitative research with migrant groups in the UK suggests community-focused screening models, which are proactive and work closely with community organisations, will be best placed to break down the barriers migrants face accessing screening.^24^

The aim of this pilot study was to co-design and evaluate a community-based screening initiative for CD in London, based on point of care testing, followed by patient management and family screening.

## Methods

### Study design & setting

This was a cross-sectional, observational pilot study, using questionnaires and point of care tests in non-healthcare settings, such as community centres, charity premises, restaurants and cultural events. This was a pilot study as it was the first time that: systematic screening had been offered for CD in the UK (aside from blood bank screening): CD POCTs had ever been used in the setting; community-based research on Latin American health had been conducted; the venues where screening took place had acted as research sites. This study is reported following STrengthening the Reporting of OBservational studies in Epidemiology (STROBE) guidelines for cross-sectional studies.^25^

### Eligibility criteria

Adults (aged ≥18 years) born in, or whose mothers were born in, the 21 CD-endemic countries of Central America, South America and Mexico (Argentina, Belize, Bolivia, Brazil, Chile, Colombia, Costa Rica, Ecuador, El Salvador, French Guiana, Guatemala, Guyana, Honduras, Mexico, Nicaragua, Panama, Paraguay, Peru, Suriname, Uruguay and Venezuela) were eligible to participate. There were no exclusion criteria other than age.

### Patient and public involvement

This study was supported by a patient and community advisory group, as well as Latin American organisations’ representatives, which was involved at both the design stage of this research, and in delivering the research. Online discussions on CD awareness, knowledge and access to care were crucial to refine community engagement and screening strategies. Point of care tests were endorsed by the advisory group, as were specific social media channels and screening event venues.

### Recruitment

Screening events (lasting between 2 and 4 hours) were organised by the study team led by NE, in collaboration with community and charity partners. Events were advertised on social media and by word-of-mouth. At these events, Spanish-speaking healthcare professionals from the UK Chagas Hub (including doctors, nurses and midwives) individually discussed the study information leaflet with eligible participants and obtained written informed consent from those willing to take part. At some events, local Latin American businesses and organisations provided refreshments and entertainment, such as musical or dance performances, paid for by the research budget.

### Data collection and sampling

**Figure 1:**
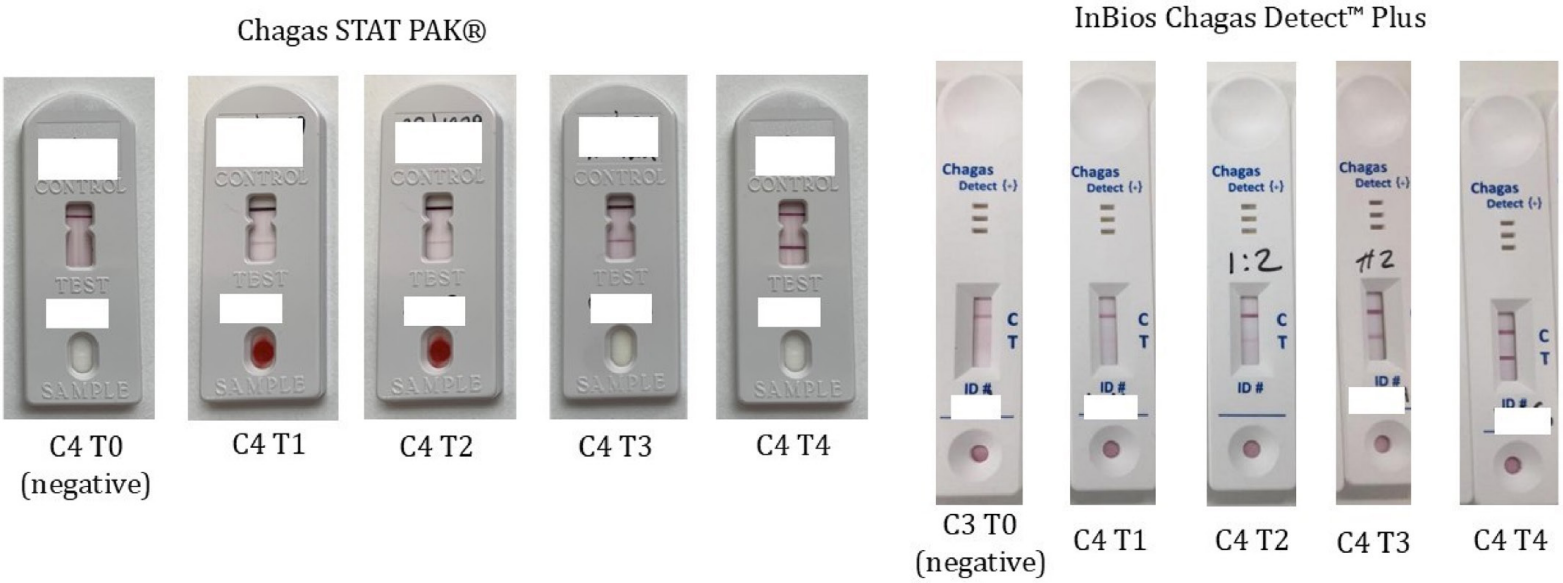
Point of care test reading charts developed by the study team. Control (C) and test (T) band strength were visually graded from 0–4 (0=negative, 1=very weak, 2=weak/moderate, 3=moderate/strong, 4=very strong). T1/2/3/4 were all considered reactive tests, requiring laboratory and clinical follow-up.

An online questionnaire (available in English, Spanish and Portuguese), conducted in the Open Data Kit Collect application,^26^ was used to capture demographic information and risk factors for CD (including age, sex, country of birth, mother’s country of birth, family history of CD and previous CD testing and treatment). A finger-prick lateral flow (capillary) blood test was used for qualitative detection of IgG to *T. cruzi*. The InBios Chagas Detect Plus (CDP) test was used initially until the supplier was unable to provide further kits, after which the Chembio Chagas STAT PAK Assay was used. The tests were performed according to manufacturer’s instructions and required a capillary blood sample to be added to the test strip, a buffer solution to be applied, and then results read in 10 (Inbios) or 15 (Chembio) minutes. All screening tests were visually examined by two trained healthcare professionals. For positive tests, a visual scale grading the strength of the band was developed by the study team (figure 1).

### Participant follow-up

**Figure 2:**
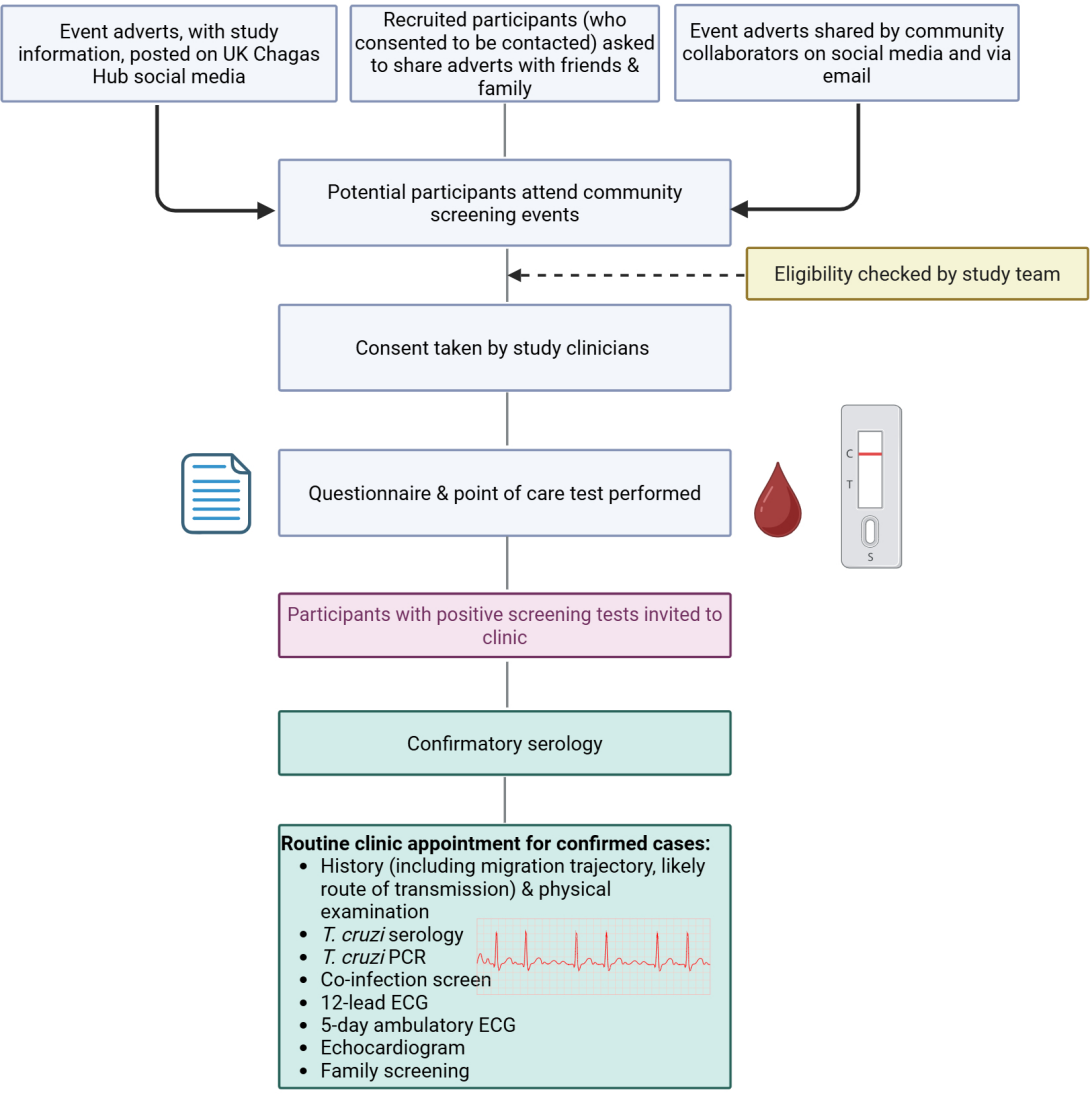
Flowchart of participant recruitment and study activities. *T. cruzi*, *Trypanosoma cruzi.*

A single positive POCT was considered a positive screening test. Participants were debriefed one-to-one in a private area by a Spanish-speaking clinician with specialist experience in CD, where the implications of the positive result were discussed. Participants were asked which form of communication (telephone call, text message, WhatsApp, email) was preferred for contact by the study team to facilitate follow-up. This was followed by referral for confirmatory laboratory investigation: the Wiener Chagatest recombinante v.4.0 ELISA, and an in-house indirect immunofluorescence antibody test (IFAT) at the Hospital for Tropical Diseases (HTD) Parasitology Laboratory. CD cases were confirmed if both the ELISA and IFAT were positive, and participants were then invited to a specialist CD clinic for further management. This included qualitative *T. cruzi* PCR at the Diagnostic Parasitology Laboratory at the London School of Hygiene & Tropical Medicine, co-infection screening, ECG, echocardiography, family screening and consideration of trypanocidal therapy (with benznidazole or nifurtimox). If symptoms suggestive of gastrointestinal involvement were reported, then imaging and referral to a specialist, as clinically appropriate, was initiated. Reminder messages before clinic appointments were sent by participants’ preferred means of communication. Figure 2 depicts the participant flow from recruitment to study activities and clinical follow-up.

### Data analysis

Point of care screening, followed by confirmatory serology, resulted in binary classification of the primary outcome (*T. cruzi* seropositivity). Prevalence of seropositivity was calculated overall, by event type and in risk groups (ie, stratified by age, sex and country of birth). The positive predictive value of the screening tool was calculated by dividing the number of true (ELISA and IFAT) positives by the total number of positive tests. The screening yield (number needed to screen to diagnose one case) was calculated (as number of people screened divided by confirmed cases) overall and by event type.

Categorical data were presented as numbers and proportions. Means and SD were calculated for normally distributed continuous variables, with medians and IQRs provided if data were not normally distributed. 95% CIs were calculated for prevalence estimates. χ² tests were used to compare categorical variables, and the Student’s t test was used for normally distributed continuous variables. Results were considered statistically significant if the two-tailed p value was less than 0.05. All data analysis was performed in STATA version 18 (StataCorp LLC, College Station, Texas).

## Results

### Community engagement and screening strategy development

Eight online consultations (with 3–10 participants each) were conducted in Spanish with a combined total of 50 members of the Latin American community who had responded to social media adverts. The most commonly cited obstacles to accessing healthcare were language barriers, difficulty booking primary care appointments and working hours precluding attendance to weekday appointments. People from endemic regions of Bolivia had heard of CD, but most other participants had not. Awareness that people living in the UK could have *T. cruzi* infection was universally low, as was awareness of any risk of vertical transmission. Participants favoured and recommended the use of social media channels (namely Facebook and WhatsApp) to raise awareness of CD and to promote screening. Screening using point of care tests was deemed acceptable to participants and was generally preferred to venepuncture. Non-healthcare settings, in localities of London with large Latin American communities, were proposed as sites for potential screening. A range of Latin American businesses and charities were suggested as potential collaborators, many of whom became involved in this study. Social media messaging was recommended to prevent loss to follow-up.

### Participant characteristics

A total of 334 individuals participated in screening across nine events in London between December 2021 and May 2023 (table 1). Five were CD-specific screening events with social media advertising (number screened=254, 76% total population screened). Four were stalls set up at Latin American community events or businesses (n=80, 24%).

**Table 1:** Characteristics of people screened in the community for Chagas disease in London, 2021–2023

	All	CD-specific event	Other event	P value*
N	334	254	80	
Age at screening, mean (SD)	43.5 (11.8)	43.9 (11.7)	42.1 (12)	0.2
Female sex, n (%)	223 (67%)	163 (64%)	60 (75%)	0.07
Female aged ≤45, n (%)	134 (60%)	91 (56%)	43 (72%)	<0.05
Country of birth, n (%)				
Argentina	6 (1.8%)	5 (2%)	1 (1%)	<0.001
Bolivia	206 (61.7%)	186 (73.2%)	20 (25%)	
Colombia	36 (10.8%)	14 (5.5%)	22 (28%)	
Ecuador	55 (16.5%)	28 (11%)	27 (34%)	
El Salvador	<5 (<1.5%)	0	(<5 (<6.3%)	
Honduras	<5 (<1.5%)	<5 (<2%)	<5 (<6.3%)	
Mexico	<5 (<1.5%)	<5 (<2%)	<5 (<6.3%)	
Nicaragua	<5 (<1.5%)	<5 (<2%)	0	
Paraguay	<5 (<1.5%)	<5 (<2%)	0	
Peru	9 (2.7%)	6 (2.4%)	<5 (<6.3%)	
Venezuela	5 (1.5%)	5 (2%)	0	
Other	7 (2.1%)	6 (2.4%)	<5 (<6.3%)	
Risk factors for CD				
First-degree relative with CD, n (%)				
Yes	119 (36%)	111 (44%)	8 (10%)	<0.001
No	160 (48%)	106 (42%)	54 (68%)	
Not known	55 (16%)	37 (15%)	18 (23%)	
Previous test for CD, n (%)				
Yes	74 (22%)	68 (27%)	6 (8%)	<0.001
No	250 (75%)	177 (70%)	73 (91%)	
Not known	10 (3%)	9 (4%)	1 (1%)	
If tested, previous test result, n (%)				
Positive	38 (51%)	38 (56%)	0	<0.01
Negative	21 (28%)	16 (24%)	5 (83%)	
Not known	15 (20%)	14 (21%)	1 (17%)	
If positive, previous treatment for CD, n (%)				
Yes	21 (55%)	21 (55%)	N/A	
No	16 (42%)	16 (42%)	N/A	
Not known	1 (3%)	1 (3%)	N/A	

For risk factors for CD, the ‘Not known’ category includes participants who did not answer that question, in addition to those who indicated they were unsure in the questionnaire. No participants reported a previous positive test result and so the question about treatment was not asked.

Country of birth groups with fewer than five participants have been concealed (to <5) to prevent identification.

* P value refers to statistical tests assessing differences between attendees at CD-specific events and those at other events (T test for mean age and χ² test for all other categorical variables).

CD, Chagas disease; N/A, not applicable.

Of the 334 people screened, 223 (67%) were women, of whom 134 (60%) were women of reproductive age (aged 15–44 years). The majority of participants (n=206, 62%) were born in Bolivia, followed by Ecuador (n=55, 17%), Colombia (n=36, 11%), and fewer than 10 each from the other countries represented (table 1).

Participants screened at CD-specific events were similar in age and sex to those screened at other events but differed by country of birth and CD-risk profile. A higher proportion of CD-specific event attendees were born in Bolivia, had first-degree relatives with CD and had previously been tested and/or diagnosed and treated for CD, compared with attendees at other (non-CD-specific) events (table 1).

### Serological confirmation of CD

A total of 79 out of 334 participants (24%) screened positively. Of the 77 participants (95%) who attended for confirmatory laboratory serology, 70 (21% total population screened) were confirmed as true CD cases (both ELISA and IFAT positive). There was no discordance between the confirmatory ELISA and IFAT. True positive CD cases differed from those with a negative POCT by CD-risk factor profile, more frequently reporting first-degree relatives with CD and previous testing for CD (table 2). Table 3 outlines the prevalence of Chagas disease in populations screened in this study, stratified by age, sex and country of birth.

**Table 2:** Risk factors for Chagas disease (CD) by point of care test (POCT) result, London 2021–2023

	Negative POCT (n=255)	Positive POCT (and confirmed true case, n=70)	P value
First-degree relative with CD, n (%)			
Yes	72 (28%)	45 (64%)	<0.001
No	137 (54%)	17 (24%)	
Not known	46 (18%)	8 (11%)	
Previous test for CD, n (%)			
Yes	29 (11%)	44 (63%)	<0.001
No	220 (86%)	24 (34%)	
Not known	6 (2%)	2 (3%)	
If yes to above, previous test result, n (%)			
Positive	2 (7%)	36 (82%)	<0.001
Negative	20 (69%)	1 (2%)	
Not known	7 (24%)	7 (16%)	
If positive result reported above, previous treatment for CD, n (%)			
Yes	1 (50%)	20 (56%)	0.95
No	1 (50%)	15 (42%)	
Not known	0 (0%)	1 (3%)	

CD, Chagas disease; POCT, point of care test.

**Table 3:** Prevalence of Chagas disease in populations tested through community screening in London, 2021–2023

	Prevalence (confirmed cases/population screened, 95% CI)
Total population screened	21% (70/334, 95% CI 17 to 26)
Screened at CD-specific event	27% (68/254, 95% CI 21 to 33)
Screened at other event type	3% (2/80, 95% CI 0.3 to 9)
Age-specific prevalence	
18–29	2% (1/43, 95% CI 0.05 to 12)
30–39	20% (16/79, 95% CI 12 to 31)
40–49	23% (26/114, 95% CI 15 to 32)
50+	40% (27/68, 95% CI 28 to 52)
Male	22% (24/111, 95% CI 14 to 30)
Female	21% (46/223, 95% CI 16 to 27)
Female aged <=45*	16% (21/134, 95% CI 10 to 23)
Country of birth	
Argentina	0% (0/6, 95% CI 0 to 46)
Bolivia	33% (69/206, 95% CI 27 to 40)
Colombia	0% (0/36, 95% CI 0 to 10)
Ecuador	0% (0/55, 95% CI 0 to 6)
El Salvador	0% (0/1, 95% CI 0 to 98)
Honduras	0% (0/3, 95% CI 0 to 71)
Mexico	0% (0/4, 95% CI 0 to 61)
Nicaragua	0% (0/1, 95% CI 0 to 98)
Paraguay	100% (1/1, 95% CI 25 to 100)
Peru	0% (0/9, 95% CI 0 to 34)
Venezuela	0% (0/5, 95% CI 0 to 52)
Other	0% (0/7, 95% CI 0 to 41)

* Women of reproductive age are presented separately as a priority group for screening (because administration of antiparasitic treatment in this population almost eliminates the risk of vertical transmission of *Trypanosoma cruzi* in future pregnancies.

CD, Chagas disease.

Two additional participants had a negative POCT but reported previous diagnosis of CD and so were linked into care for follow-up (one of whom was confirmed as a true positive, one negative on confirmatory serology).

### Point of care test characteristics

A total of 168 participants were screened with the CDP Rapid Test POCT alone, 116 with the Chembio Chagas STAT PAK Assay alone, and 50 with both. Discordance between the two POCT results was seen in two out of 50 participants who had both POCTs (one CDP positive, STAT PAK negative and vice versa, both with weakly reactive test lines). One of these participants attended for confirmatory serology which was negative (both ELISA and IFAT negative) while the other was lost to follow-up.

The overall positive predictive value (of either POCT used throughout this study) was 91% (70 laboratory-confirmed positives out of 77 positive POCTs). For the CDP alone, it was 83%, for STAT PAK alone, it was 97%, and for both combined, it was 92% (with either positive interpreted as a positive screen).

Of the 70 true positive cases (POCT positive, subsequent laboratory ELISA/IFAT positive), five (7%) were visually assessed to have a very weak test line (graded 1 out of 4), 17 (24%) a weak-moderate test line (graded 2 out of 4), 35 (50%) a moderate-strong test line (graded 3 out of 4) and 13 (19%) a very strong test line (4 out of 4) (figure 1).

Of the seven false positive cases (POCT positive, subsequent laboratory ELISA/IFAT negative), all seven were visually assessed to have a very weak POCT test line (graded 1 out of 4). The potential of cross-reaction to explain the false positive tests was investigated by serological screening for HIV (0/7, 0% positive), strongyloidiasis (2/7, 29% positive), hepatitis B (1/7, 14% anti-hepatitis B core positive), hepatitis C (0/3, 0%), human T-lymphotropic virus 1 (0/1, 0%) and syphilis (0/2, 0%).

### Linkage to care

Of the 81 people requiring follow-up (79 positive POCTs plus two with negative POCT who reported previous positive testing), 78 (96%) attended the tertiary hospital for confirmatory blood testing and 75 (93%) attended the specialist Chagas clinic appointment for consultation and ECG. Of the 70 true confirmed cases of CD detected through this community pilot, 63 (90%) remained engaged in follow-up as of January 2025 (mean duration of 2.5 years follow-up since screening). All participants requiring follow-up indicated a preference for WhatsApp as the communication means to facilitate follow-up appointments.

### Screening yield

The number needed to screen to detect one confirmed case (linked into care) was 5.2 overall, 3.7 at CD-specific events and 40 at non-CD-specific events.

The number needed to screen to link one (previously untreated) person into care for antiparasitic therapy was 8.6.

The number of women of reproductive age needed to screen to link one confirmed case into care was 6.4.

### Clinical assessment

After clinical assessment and baseline investigations (ECG, ambulatory ECG and echocardiogram) of the 70 cases of confirmed CD, 18 (26%) were *T. cruzi* PCR positive (one of whom reported previous trypanocidal treatment).

Eight (11%) were classified as having determinate disease (seven with cardiac and one with digestive involvement), of whom four were PCR positive and four PCR negative.

### Co-infection screen

Co-infection was investigated by serological screening for HIV (0/67, 0% reactive), strongyloidiasis (9/69, 13% reactive, of which four were borderline weakly reactive ELISAs of uncertain clinical significance), hepatitis B (1/22, 5% anti-Hb core positive), hepatitis C (0/23, 0%), HTLV-1 (0/18, 0%) and syphilis (1/3, 33% reactive, treated latent syphilis).

### Management

39 people (of 45 previously untreated, 87%) with CD detected through this community screening were treated with antiparasitic therapy (35 with benznidazole, four with nifurtimox).

### Family screening

33 people with CD detected through this community screening (47%) were mothers to children living in the UK (all of whom were offered serological screening as part of routine care). 41 family members were screened and *T. cruzi* infection was detected in one child who was subsequently linked into care and treated with antiparasitic therapy (1/41, 2.4% case detection through family screening).

## Discussion

This study represents the first community-based screening initiative for CD, and the largest population screened to date, in the UK. Over one in five people screened were confirmed to have *T. cruzi* infection. Collaborating with Latin American charities and community groups (and coproducing outreach strategies) effectively reached populations with a large burden of CD, predominantly people from endemic parts of Bolivia, and resulted in high linkage to care.

It is important to interpret the high seroprevalence of CD detected in this study (21%) in the context of published data from other non-endemic settings. Laboratory-based screening programmes for CD exist in many such settings, such as Spain,^27^,^32^ Italy^33^ and Canada.^34^ In a meta-analysis of CD seroprevalence studies performed throughout Europe, the pooled prevalence overall was 4.2% (95% CI 2.2% to 6.7%), but differed by setting—being highest (8.7%, 95% CI 7.7% to 9.9%) in primary care/community settings.^35^

Community-based approaches, where venepuncture is performed in the community but samples processed in laboratories, have been piloted in European cities such as Barcelona, Rome, Alicante and Paris (where the reported *T. cruzi* seroprevalences were 9%, 9%, 11% and 24% respectively).^36^,^39^

Despite use in endemic countries,^22 23^ POCT-based screening for CD has been used less commonly in non-endemic settings. *T. cruzi* POCTs were first used in Switzerland for testing migrants and returning travellers, both in healthcare settings and at community events.^40^ 1010 adults were tested in total, of whom 84 (8%) were at community events. The overall prevalence was 1.6% (and 0% in travellers); however, prevalence was much higher (15.5%)—and similar to that found in this study—when POCTs were offered at community events. In a recent pilot study of POCTs in 19 pharmacies in Barcelona, two out of 64 (3%) of participants screened positively.^41^ Due to low uptake in the early stages of this pharmacy project, it is recommended that its acceptability is assessed in other contexts prior to adoption. As with this London-based study, these examples of community-based screening do not reach populations representative of the entire migrant communities; rather, they are demonstrably effective at reaching high prevalence groups.

The main strengths of this study lie in its culturally informed recruitment strategy (developed through an extensive programme of patient and public involvement) which resulted in high engagement of Latin American migrants, a population historically under-represented in research and underserved by the healthcare system,^42^ and a very high screening yield. The use of POCTs allowed this project to reach communities in locations that were both geographically convenient and culturally relevant. In addition to detecting new cases, community-based awareness raising and screening also linked people with previously diagnosed CD into care with minimal loss to follow-up. This pilot study also demonstrated the feasibility of some novel approaches to research activities, for example, that health professional volunteers were willing and able to perform the research activities, that eligible participants do attend screening events advertised on social media and that venues such as community centres are suitable research sites.

Although the results of this study provide some insight into the prevalence of *T. cruzi* infection in the community tested, its main limitations relate to the opportunistic sampling frame, meaning that prevalence estimates are not generalisable to the whole Latin American migrant population. In this study, non-Bolivians were under-represented and Brazilians were not represented at all. It is recommended that community engagement and screening for CD is expanded to include more representative populations, and the inclusion of children in these initiatives should be considered. Community engagement with Brazilians (conducted in Portuguese), who comprise the largest group of Latin Americans in London, is recommended to understand this community’s specific barriers to accessing CD screening. A further limitation is that this study did not collect data on migration status. Given that a study of aggregate data from published literature and surveillance sources in Europe showed a very high prevalence of CD in undocumented migrants (on average 45% of total expected cases),^43^ this is an important gap. Further research is recommended to understand and address the barriers to diagnosis for the most underserved groups, such as undocumented migrants, labour migrants, refugees and asylum seekers. Multi-disease screening for Latin American migrants in the UK should also be considered for broader impact.

Although the Swiss healthcare system differs from the UK context, many of the challenges and strategies to address the underdiagnosis of CD outlined in a recent review article^44^ are similar to those cited in the UK literature.^12 13^ For example, at-risk blood donors have been screened for many years, but there is no formal antenatal screening of at-risk pregnant women, a group whom we regard as a priority for screening. Systematic and mandated screening for CD in target populations in the UK is recommended to reach the populations at risk. Enhanced surveillance and inclusion of CD as a notifiable disease, alongside work to reduce the systemic barriers to healthcare that migrants face, are needed to better understand CD epidemiology and ensure that screening initiatives do not widen existing health inequalities.

## Conclusion

Community-based awareness-raising and point-of-care screening can inform understanding of Chagas disease epidemiology, reach populations with high prevalence in non-endemic settings and—importantly, link positive cases into care.

## Data Availability

All data relevant to the study are included in the article or uploaded as supplementary information.
